# Concerns of Indian Mothers with Children Having Severe-to-Profound Hearing Impairment at Diagnosis and after 1–3 Years of Therapy

**DOI:** 10.1155/2012/593405

**Published:** 2012-08-05

**Authors:** Nachiketa Rout, Megha Khanna

**Affiliations:** Department of Speech, Hearing and Communication, National Institute of Empowernment of Persons with Multiple Disability (NIEPMD), ECR, Muttukadu, Chennai 600041, India

## Abstract

Counseling training in graduate programs continues to be underrepresented. If parental queries are not addressed adequately, they keep visiting one doctor after another. *Objective*. The aim of the study is to identify maternal needs of children with hearing impairment at two stages of habilitation, that is, just after diagnosis (group I) and after receiving 1 to 3 years of language therapy (group II). *Methods*. Two groups of mothers were asked to speak their queries about aural habilitation of their children. Queries were recorded, summarized, and categorized as per their priorities. *Results*. Group I mothers wanted to know about how the child would learn to listen and speak (45%), causes of hearing loss (33.7%), understanding the ear and hearing (10.2%), understanding the audiogram (7%), and coping with emotional aspects of hearing loss (5%), while group II parents had priorities concerning speech development (24.5%) followed by child independence and employment (17.3%), schooling (15.6%), problem behaviors (11%), amplification device (9.4%), duration of therapy (8%), future of the child (8%), and questions about how can my child get adjusted to the “normal” world (6%). *Conclusions*. Culture- and language-specific materials to explain these issues need to be developed.

## 1. Introduction

Apt counseling may have a direct impact on the success of early hearing detection and intervention programs along with reducing the negative effects of permanent hearing loss [1–3].

 The clinician is confronted with a difficult task during the first few contacts with the parents because they are in a state of shock and they seek help in changing the child's behavior so that their anxiety can be dissipated [4]. When asked, patients too frequently report that audiologists do not seem to understand their difficulties [5, 6]. This may puzzle audiologists, because, as educated professionals, they feel that they “do deeply” understand their patient's difficulties. The breakdown may occur because of a mismatch in communication intent; that is, although the audiologist's responses are not wrong, they do not match the intent of the patient's remarks [7]. English et al., 1999 suggested that this communication mismatch may be a natural consequence of graduate training, whereby student success is measured in terms of adequately explaining high-level technical information to instructors and supervisors. 

In India, it is in part is also because of the undue influence of the medical model of treatment in dealing with permanent hearing loss (HI). Audiologists are influenced by the medical model and view HI as a “disease” to be cured, rather than a “condition” with which the child and the family have to live with for the rest of their life. The audiologist many times feels there job is done with the diagnosis of the hearing loss and the prescription of the hearing aid. Counseling involves briefing out the findings of the hearing loss and the control switches of a hearing aid. If the counseling is done keeping view of the social model, the parents and family needs are considered first and the information is provided depending upon their priorities, rather than briefing findings which seem to be important to the counselor. There has been a recurring emphasis on the need for more empathetic behavior by professionals and the need for them to have a greater understanding of the emotional impact of the diagnosis on the family, which does not receive due attention [4, 8–10]. It has also been suggested that difficulties experienced by hearing-impaired persons often have their roots in parent attitudes towards the handicap [11]. Counseled patients wore their hearing aids more and achieved greater reduction in perceived hearing handicap than noncounseled patients [12]. Studies confirmed the hypothesis that counseling training in graduate programs continues to be underrepresented and that graduates do not feel fully prepared to conduct counseling in the field of communication disorders following graduation [13].

The scenario in countries like America is quite different from India. In India a universal newborn hearing screening program is yet to be implemented at national level. Unlike in west habilitation initiatives are taken up by parents and the mean age of detection of hearing loss is about 3.03 (SD: ±1.3) years and aural habilitation begins by a mean age of 7.36 (SD: ±4.06) years [14]. Majority of India lives in rural areas under a joint family system; divorce and single parents are rare. Poor literacy, multilingualism, variety of cultural beliefs, socioeconomic conditions, and terrain are quiet diverse in India which houses 17.5% of the world's population. All the factors influence the habilitation programmes and thus place the need for culture-specific studies. 

 The aim of the study is to identify the maternal needs of children with hearing impairment at two stages of habilitation, that is, just after diagnosis and after receiving 1 to 3 years of speech and language therapy. Understanding the needs would promote developing focused counseling strategies at different stages of aural habilitation which in turn will facilitate the process.

## 2. Methodology

### 2.1. Participants

The participants of the study included two groups of mothers of children with severe-to-profound-degree of hearing loss. The mother's ages ranged from 21 to 38 years. All the mothers belonged to the middle socio economic strata with an income of less than 6500 rupees per month. All the children had bilateral severe-profound sensory neural hearing loss and had been prescribed a strong class body-worn hearing aid. The hearing aids were issued free of cost under ADIP (assistance to disabled persons for purchase/fitting of aids and appliances) scheme of Government of India (Ministry of Social Justice and Empowerment, 2009). 

Group I included forty-nine mothers in the mean age range of 31.5 years (SD: ±2.72) whose children had recently been diagnosed with hearing loss and Group II consisted of a random sample of 52 mothers with a mean age of 37.3 years (SD: ±3.62) whose children were receiving speech and language therapeutic intervention since last 1 to 3 years. All the children aged between 3 and 8 years with a mean age 4.21 (SD: ±1.13) years. The mothers in both groups had received formal education up to class Xth or more. The interview was carried out in a one-to-one setting in Bengali or Hindi language, whichever was the native language of the participant. The average time taken in an interview ranged from 20–25 minutes. Consent was obtained from the mothers after explaining to them the purpose of the study. 

### 2.2. Procedure

After explaining the aim of the study, the participants were instructed to write down queries regarding their child's habilitation. The written queries were collected and tabulated. A total of 305 queries were obtained.

- The queries of group I mothers were placed under five major categories after tabulation. The five major categories included causes of hearing loss, coping with emotional aspects of hearing loss, understanding the audiogram, learning to listen and speak, and understanding the ear and hearing
- The queries of group II mothers could be placed under eight major categories. The eight major categories included speech and language development of the child, schooling, can my child get adjusted to the “normal” world, problem behaviors, like restlessness, lack of attention, and so forth, amplification device, duration of therapy, future of the child and child independence and employment.

Statistical analysis was carried out using statistical package for social science (SPSS) for windows (version 11) software. 

## 3. Results

**Figure 1:**
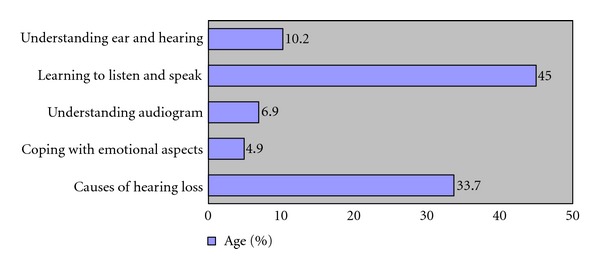
Depicting the queries put forth by group I mothers.

**Figure 2:**
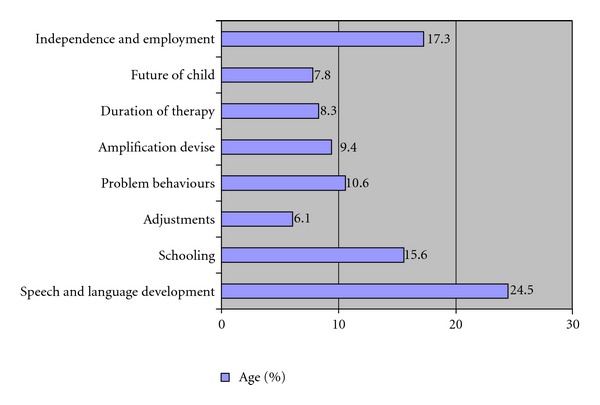
Queries of group II mothers regarding their child.

 Descriptive analysis was used to estimate the percentage occurrence of responses to each question item in each category. The categories were analyzed in terms of number of times each category was asked by the parents and was ranked accordingly. The percentage of distribution of queries and priorities occurring in each category for both the groups is listed in Figures 1 and 2.

## 4. Discussion

There are four major ways in which hearing loss affects children. It causes delay in the development of receptive and expressive communication skills (speech and language), the language deficit causes learning problems that result in reduced academic achievement, communication difficulties often lead to social isolation and poor self-concept, and it may have an impact on vocational choices [15]. Hence questions pertaining to these areas are most frequently asked. 

In the present study mothers were selected as participants because they commonly accompanied the child for therapy. Fathers went out for jobs to meet the financial needs of the family. During the first counseling session after diagnosis when both the parents were present, most of the mothers found difficulty in understanding the technicalities of the hearing aid and the hearing loss which their child had. Sweetow and Barrager (1980), via use of a questionnaire survey, attempted to study the need for the audiologist to translate complicated terminology into language easily understood by the parents and the need for written information related to hearing impairment to be dispersed was repeatedly stressed [16].

### 4.1. Group 1

A survey by Roush and Harrison [17] identified priorities of parents at diagnosis. They found that most of the parents wanted to know about the causes of hearing loss, coping with emotional aspects of hearing loss, understanding the audiogram, learning to listen and speak, and understanding ear and hearing at the time of diagnosis. In the present study mothers rated category T4 (learning to listen and speak) as their most important priority. This reflects the acceptance of the child's hearing loss on part of the mothers and an eagerness to begin a plan of action. Ali Yavar Jung National Institute for the Hearing Handicapped (Eastern Regional Center) is a tertiary center dedicated to the aurally handicapped. The institute mostly gets cases through referral from various sources like primary health centers, private hospitals, and referral by old patients, and so forth. Most of the caregivers and clients come with a complaint of hearing loss and speech difficulty hence the diagnosis of hearing loss mostly does not shock them. The clients are much concerned about knowing the exact degree and type of hearing loss, obtaining a hearing aid, and availing a report to obtain a disability certificate. Parents counseling in such tertiary centers should focus upon the importance of hearing and how the hearing aid helps the child to get exposed to speech. Many parents feel that the speech deficit is due to the restricted tongue mobility and may ask for activities to improve tongue movements. The exceeding importance of auditory training needs to be emphasized and its contribution towards learning speech step by step should be detailed by giving them activity booklets and materials in the native language which is mostly lacking in Indian languages. Parents rated causes of hearing loss as their second highest priority. Historically, only about half the cases of childhood hearing loss have had known causes [18] although the advent genetics testing is substantially reducing this number. Many parents struggle, sometimes for long time on the question of casuality. So professionals should at least explain the probable cause of the hearing loss if it cannot be specified otherwise. It should be emphasized that most of the etiologies of hearing impairment are not known. Parents should be counseled that pondering less on the etiology and concentrating more upon these rehabilitative aspects would be much beneficial as the critical age for speech development is one of the shortest. If the child is not encouraged to use regular amplification there is a very high chance that an irreversible neural reorganization will occur in the auditory pathway and the cortex. Later even if the best hearing aids are fit the child's nervous system cannot appreciate its output.

 Understanding the ear and hearing was another frequently stated category and has been ranked as third priority. Even though parents may differ in the level of information they desire, almost all are interested in knowing at least basics about the structure and function of the ear. Simple diagrams and models are most helpful in explaining the various parts of ear. Working of the ear and its parts can be best explained through analogies. Analogies of the funnel are found useful in explaining the function of the external ear, the external ear collects sounds and sends them to the middle ear. The middle ear has an ear drum and three bones which pass the sound into the inner ear. Tympanic membrane or ear drum is the first part of the middle ear which collects the sound and passes it to the middle ear bones. It is a common notion in India that hearing loss is due to a defective ear drum. Parents need an explaination about the ear drum and its status and its contribution if any to their child's hearing loss. The middle ear functions like a voltage stabilizer which mostly increases the sound pressure level for better hearing and protects the ear from loud sounds by decreasing the loud sounds before they reach the inner ear. The inner ear is like a harmonium or piano with high-frequency sound receptors basally and low-frequency receptors apically. These receptors are known as hair cells. Most of the children with sensory neural hearing loss have damaged hair cells. The hair cells code sounds into electrical impulses. These electrical impulses are carried by the auditory nerve and further by the central auditory pathway; both function like electric wires. The auditory pathway delivers the electrical impulses to the auditory cortex. The auditory cortex decodes the electrical impulses into meaningful information and the sound is perceived.

 Once the normal functioning of the ear is explained using the analogies, parents can be pointed to the area which is damaged for their child and how the intervention strategy (like a hearing aid) would help the child. 

**Figure 3:**
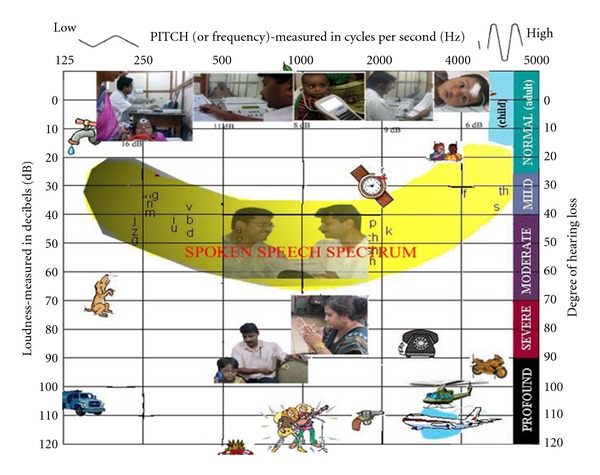
Depicting the sound intensities of common sounds along with the degree of hearing impairment. It also depicts Maximum Permissible Ambient Noise Levels (in dB re: 20 micro pascals) inside an audiometric test chamber ventilation fan on.

The fourth priority category was understanding the audiogram. Parents may be unable to fully understand their child's hearing loss, as shown by an audiogram. Acknowledgment by the audiologist that the audiogram is abstract and difficult to comprehend may cause parents to feel more uncomfortable asking questions. Moreover, it is important to recognize that most families and parents are not interested in the audiogram per se, but rather what it can tell them about their child's hearing. An audiogram with a speech banana on it along with the intensities of various commonly found sounds in the environment helps parents understand their child's hearing. A portable sound-level meter is also found to be useful in helping the parents understand the various intensities of common sounds like name call, clap, door knock, telephone ring, and so forth. The average sound levels in the sound-treated rooms can also be included to give them an idea about the “quietest” environment (Figure 3).

Coping with emotional aspects of hearing loss was also selected as an important category and was ranked as fifth information priority by mothers. There has been a recurring emphasis on the need for more empathetic behavior by professionals and the need for them to have a greater understanding of the emotional impact of the diagnosis on the family [19]. This is a domain which is most often missed by the newly trained rehabilitation professionals who often wonder why parents take so long to accept that their child has a hearing loss or the importance of regular use of hearing aid. Many professionals may get annoyed by parents who try to test and retest to get the hearing loss confirmed. A diagnosis of hearing loss is life changing for many families. A professional through effective counseling can help parents pass through the different stages like grief denial, anger, bargaining, and acceptance without adversely affecting the outcomes of rehabilitation. 

### 4.2. Group 2

A few months after diagnosis of hearing loss, parents' priorities were all related to developing their child's ability to communicate effectively and to early-intervention services [17]. In the present study, after 1–3 years of availing therapy, the first category, “speech and language development of the child,” was ranked as the first priority by the mothers. Probably the gain in linguistic skills by children was not as per the mothers expectations. It is a common quote in India “the first day of fitting hearing aid the audiologist is happy, by six months the mother is happy and it takes a year to make everybody happy.” A significant improvement in the functional communication was noticed by parents after body level hearing aid was used for 12 months and more [20]. Thus it is essential on part of the audiologists and the speech language pathologists to counsel the parents regarding the expected outcomes of therapy during the intervention programme and the various factors influencing it. Developing parents as cotherapists by demonstrating for them the short-term goals to be carried at home helps reduce parental stress, increases the therapeutic impact, and gives them a lot of confidence. 

The second priority was the query about the child independence and employment and, “future of the child” was the seventh priority. Parents need to be briefed about the various provisions provided to the individuals with hearing impairment (HI) by the Government of India [21]. The Government of India has identified about 2000 jobs for the individuals with HI. The details can be found in the web page of the Chief Commissioner of Disabilities, India (http://www.ccdisabilities.nic.in/). The identified jobs are reviewed every 3 years and new jobs are added to the list. About 1% government jobs are reserved for the individuals with HI. The NHFDC (National Handicapped Finance and Development Corporation) provides financial assistance to individuals with HI ranging from 6,500 rupees to 25 lacks to start to develop their own business. There are 3% reservations for the disabled population in all educational institutes including training centers and technical training institutes. Individuals with HI can avail a travel train concession of 50% along with one escort who also gets the same benefit. 

The second category “schooling” was ranked as the third priority by the mothers. It has been reported that parents want information about the full range of intervention options and ramifications of the hearing loss on education [22]. Children with severe-to-profound hearing loss usually achieve skills no higher than the third- or fourth-grade level, unless appropriate educational intervention occurs early. The gap in academic achievement between children with normal hearing and those with hearing loss usually widens as they progress through school. The level of achievement is related to parental involvement and the quantity, quality, and timing of the support services children receive [15]. In India, most of the children are referred to the nearest government run regular school under the Sarva Shiksha Abhiyan (education for all movement). Sarva Shiksha Abhiyan since 2001 aims to provide useful and relevant education to all children including those with disabilities. A special educator attends the school twice weekly as a resource teacher and helps children to cope up with their educational curriculum. However there are various ground-level difficulties; at present remedial education is still not available in many places even in metro cities like Mumbai [23] and in Kolkata. Most of the parents cannot afford the services of a special educator who may charge 100 to 250 rupees per-session or a speech therapist whose per-session charges are still high. The Sarva Sikha Abhayan admits children aged six years and above [24]; by this age the critical period of speech and language development is towards its end. Children below five years of age are referred to the Anganwadi and centers run by the Government of India since 1975. The main reason behind the formation of anganwadis was the high infant mortality rate (IMR) in India [25]. The anganwadi workers are neither trained nor equipped to facilitate speech and language development of a child with hearing impairment so the child's development gets neglected in these areas.

The fourth category “problem behaviors like restlessness, lack of attention, and so forth” was rated as the 4th priority by the mothers. Restlessness, one of the most commonly seen problem behaviors in children with hearing loss, can mostly be attributed to their impaired communication skills. Children with severe-to-profound hearing loss often tend to become more physical rather than being verbally persuasive. For instance a 3-4-year-old child often persuades his/her peer with a toy which he/she needs; the better the child can verbally persuade the easier it is for him/her to obtain the toy without snatching it. On the other hand a deaf child of the same age may ask for the toy using gestures and/or some rudimentary commands and may not be capable of convincing the peer; consequently he/she may try to physically snatch the toy. Similarly the child with hearing impairment may miss the classroom discourse and lose interest in the classroom activities and engage in “actions” which are labeled as problem behavior by the teacher. 

 “Amplification device” was rated as the fifth priority by the mothers. All the participants in the study had been dispensed a body-level hearing aids; however the parents wanted to know about the efficacy of the behind-the-level hearing aids, the digital aids, and the advantages of the cochlear implant for their child. Apart from the cosmetic appeal, parents often find it easy to understand the advantage of the behind-the-ear hearing aid when the concept of localization and the fact that the “ear” is placed closer to the ear in the behind the-ear-hearing aids is explained to them. Digital signal processing and its advantage are quiet easily understood if parents receive an explanation about the capability of a digital aid to manipulate and modify the incoming signal as per the listening needs of the user. Thus the digital processing of the incoming signal helps to reduce noise, enhance speech signal, and the hearing aid automatically changes the strength and the direction of the amplification based on the source of noise. There are additional facilities of in situ measurements. Hearing aids should be preferably purchased from outlets which offer good after-sales service.

 Cochlear implants are gaining popularity in India. Mothers are quiet curious about the cochlear implants. However mothers need to be explained that not all severe-to-profound hearing impaired children are candidates for cochlear implants. A cochlear implant is used only when children fail to develop simple auditory skills after consistently using binaural hearing aids along with auditory training for a period of six months. Many parents feel that the cochlear implant unlike a hearing aid is completely implanted/invisible and returns back the child's audibility to “normal.” It should be elaborated that the child needs regular intensive habilitation and recurring cost of speech and language therapy even after implantation.

The sixth priority was the category “duration of therapy.” A general guideline of time duration for achieving target speech behaviors can be obtained from the developmental tool for speech and language development. For instance, if a “normally” developing child takes 6 months to more from one-word to phrase level a child with hearing impairment will take a minimum of six-month time. But as the child has an impaired auditory system, which even after being aided does not give the same sound perception as a un impaired ear thus the child takes a longer. The progress of the child is influenced by other factors like hearing age of the child, the amount of aided benefit if the presence of additional handicapping conditions, and so forth.

The third category “social adjustment” was ranked as the eighth and last priority across all the interviewed mothers. Children with severe-to-profound hearing losses often report feeling isolated, without friends, and unhappy in school, particularly when their socialization with other children with hearing loss is limited. These social problems appear to be more frequent in children with mild or moderate hearing losses than in those with a severe-to-profound loss [15]. In India a child with hearing impairment from rural areas is much easily accepted and adjusted as compared to the urban child. The urban society and parents have higher expectations from a child as compared to a rural child who faces lower educational, vocational, and social sophistication demands. Al-Saaidah, Mazahreh, Al-Farah, and Al-Kharabsheh in 2010 found that the social adjustment level of the hearing impaired was high [26]. Furthermore, results have shown that there are differences of social adjustment level which could be attributed to the severity of the impairment. The more the hearing loss poorer was adjustment however no significant differences were found in social adjustments based upon the student's age. In India many mothers avoid to attend social functions and prevent their child to do so because of the various questions put forth by people. 

## 5. Conclusion

Counseling sessions should help mothers to answer questions like, what is wrong with the child, will he wear the hearing aid for his entire life does he have intelligence as his peers, will “normal” children get affected if they play with the HI child, the child does not respond when called from a distance even after putting the hearing aid what is the use of the hearing aid, why does he talk like this, can he support himself and his family when he/she grows old, can she ever get married, and so forth. The mothers need to be explained how to describe the peers about the hearing aid, how it helps the child and why it should not be played with.

## References

[1] Fitzpatrick E, Angus D, Durieux-Smith A, Graham ID, Coyle D (2008). Parents’ needs following identification of childhood hearing loss. *American Journal of Audiology*.

[2] Crandell CC, McDermott DJ, Pugh K (1996). A survey on amplification and counseling skills training in audiology. *The Hearing Review*.

[3] Luterman D, Kurtzer-White E (1999). Identifying hearing loss: parents’s needs. *American Journal of Audiology*.

[4] Webster EJ (1966). Parent counseling by speech pathologists and audiologists. *Journal of Speech and Hearing Disorders*.

[5] Glass L, Elliot H (1992). The professionals told me what it was but that was not enough. *Shhh*.

[6] Martin FN, Krall L, O’Neal J (1989). The diagnosis of acquired hearing loss. Patient reactions. *ASHA*.

[7] English K, Mendel LL, Rojeski T, Hornak J (1999). Counseling in audiology, or learning to listen: pre- and post-measures from an audiology counseling course. *American Journal of Audiology*.

[8] Roush J, Seewald RC (2000). Implementing parent-infant services: advice from families. *A Sound Foundation through Early Amplification: Proceedings of an International Conference*.

[9] Mindel (1971). *They Grow in Silence: The Deaf Child and His Family*.

[10] McCormick B (1975). Parent guidance: the needs of families and of the profession worker. *Teacher of Deaf*.

[11] Hedgecock LD, Noland RL (1971). Counselling children of accousticaly handicapped children. *Counsilling Parents of the Handicapped*.

[12] Brooks DN (1979). Counselling and its effect on hearing aid use. *Scandinavian Audiology*.

[13] Phillips (2008). Counseling training in communication disorders: a survey of clinical fellows. *Contemporary Issues in Communication Science and Disorders*.

[14] Rout N, Parveen S, Chattopadhyay D, Kishore MT (2008). Risk factors of hearing impairment in Indian children: a retrospective case-file study. *International Journal of Rehabilitation Research*.

[15] American Speech and Hearing Association (2011). *Effects of Hearing Loss on Development*.

[16] Sweetow RW, Barrager D (1980). Quality of comprehensive audiological care: a survey of parents of hearing-impaired children. *ASHA*.

[17] Roush J, Harrison M (2002). What parents want to know at diagnosis and during the first year. *The Hearing Journal*.

[18] Davis H (1993). *Counsilling Parents of Children with Cronic Illness or Disability*.

[19] Luterman DM, Kurtzer-White E, Seewald RC (1999). *The Young Deaf Child*.

[20] Ghosh S (2007). *Evaluating prescription of body-worn hearing aids in preschool children using Parent’s Evaluation of Oral Aural Performance of Children [dissertation]*.

[21] Government of India (1996). *The Person With Disabilities (Equal Oppurtunities, Protection of Rights and Full Participation) Act 1995*.

[22] Martin FN, George KA, O’Neal J, Daly JA (1987). Audiologists’ and parents’ attitudes regarding counseling of families of hearing-impaired children. *ASHA*.

[23] Karande S, Mehta V, Kulkarni M (2007). Impact of an education program on parental knowledge of specific learning disability. *Indian Journal of Medical Sciences*.

[24] Sarva Siksha Abhayan http://ssa.ap.nic.in/framework.html.

[25] http://en.wikipedia.org/wiki/Anganwadi.

[26] Gregory S (1976). *The Deaf Child and His Family*.

